# Shedding the Light on Post-Vaccine Myocarditis and Pericarditis in COVID-19 and Non-COVID-19 Vaccine Recipients

**DOI:** 10.3390/vaccines9101186

**Published:** 2021-10-15

**Authors:** Rima Hajjo, Dima A. Sabbah, Sanaa K. Bardaweel, Alexander Tropsha

**Affiliations:** 1 Department of Pharmacy, Faculty of Pharmacy, Al-Zaytoonah University of Jordan, Amman 11733, Jordan; dima.sabbah@zuj.edu.jo; 2 Laboratory for Molecular Modeling, Division of Chemical Biology and Medicinal Chemistry, Eshelman School of Pharmacy, The University of North Carlina at Chapel Hill, Chapel Hill, NC 27515, USA; alex_tropsha@unc.edu; 3 National Center for Epidemics and Communicable Disease Control, Amman 11942, Jordan; 4 Department of Pharmaceutical Sciences, School of Pharmacy, University of Jordan, Amman 11942, Jordan; s.bardaweel@ju.edu.jo

**Keywords:** COVID-19, myocarditis, pericarditis, SARS-CoV-2, systems biology, vaccine, VAERS

## Abstract

Myocarditis and pericarditis have been linked recently to COVID-19 vaccines without exploring the underlying mechanisms, or compared to cardiac adverse events post-non-COVID-19 vaccines. We introduce an informatics approach to study post-vaccine adverse events on the systems biology level to aid the prioritization of effective preventive measures and mechanism-based pharmacotherapy by integrating the analysis of adverse event reports from the Vaccine Adverse Event Reporting System (VAERS) with systems biology methods. Our results indicated that post-vaccine myocarditis and pericarditis were associated most frequently with mRNA COVID-19 vaccines followed by live or live-attenuated non-COVID-19 vaccines such as smallpox and anthrax vaccines. The frequencies of cardiac adverse events were affected by vaccine, vaccine type, vaccine dose, sex, and age of the vaccinated individuals. Systems biology results suggested a central role of interferon-gamma (INF-gamma) in the biological processes leading to cardiac adverse events, by impacting MAPK and JAK-STAT signaling pathways. We suggest that increasing the time interval between vaccine doses minimizes the risks of developing inflammatory adverse reactions. We also propose glucocorticoids as preferred treatments based on system biology evidence. Our informatics workflow provides an invaluable tool to study post-vaccine adverse events on the systems biology level to suggest effective mechanism-based pharmacotherapy and/or suitable preventive measures.

## 1. Introduction

Since the identification of SARS-CoV-2 virus in 2019 and the emergence of Coronavirus disease of 2019 (COVID-19) as a global pandemic, there has been an upsurge of vaccine research and development worldwide with several vaccines that completed the clinical evaluation phases and were authorized for COVID-19 [1,2]. The theoretical and technological strategies used to develop the currently approved vaccines are diverse allowing for their preferential use in distinct groups of the human population [3]. However, owing to COVID-19 vaccines’ rapid development and the innovation of the implemented technologies, it is reasonable to expect that some issues or problems, such as unexpected side effects, associated with the use of a new vaccine against a new disease may arise and would need to be addressed.

Adverse reactions to vaccines are usually trivial. The Vaccine Adverse Event Reporting System (VAERS) database provides information on reports of adverse effects after vaccination with vaccines approved for use in the United States. Recently, several worrisome reports have been released on the development of acute myocarditis after COVID-19 vaccination [4,5].

The Advisory Committee on Immunization Practices (ACIP) at the U.S. Centers for Disease Control and Prevention (CDC) has released a statement on May 17, saying that a few young vaccine recipients, predominantly male adolescents and young adults, developed a condition known as myocarditis, which is defined as the inflammatory processes of the muscular walls of the heart (myocardium) which result in injury to the cardiac muscle cells (myocytes), according to MeSH Descriptor Data 2021 [6]. Manifestations range from subclinical to sudden death. Myocarditis can reduce the heart’s ability to function or cause arrhythmia CDC [7,8]. Currently, there are increasing numbers of adverse even reports of post-COVID-19 myocarditis and pericarditis [9]. Pericarditis is inflammation of the fibro-serous sac surrounding the heart (pericardium) from various origins, such as infection, neoplasm, autoimmune process, injuries, or drug-induced according to MeSH Descriptor Data 2021 [10]. Pericarditis usually leads to pericardial effusion or constrictive pericarditis.

Myocarditis and pericarditis symptoms can range from asymptomatic, to chest pain with little or no overt cardiac damage, to acute and severe cardiac dysfunction associated high mortality if untreated [11]. These conditions has been frequently linked to common viral infections and less often to hypersensitivity related to drug reactions in the human body [12]. Thus far, the CDC did not find a causal link between myocarditis and COVID-19 vaccines [8]. 

Herein, we introduce an informatics workflow to study vaccine adverse events on the systems biology level. We also shed the light on post-vaccine myocarditis and pericarditis by providing answers to the following key questions: What COVID-19 vaccines were linked to myocarditis and/or pericarditis? What sex and age group were mostly affected? Are these adverse events affected by the vaccine dose? Are post-vaccine myocarditis and pericarditis common post-non-COVID-19 vaccines? Finally, we attempt to provide a mechanistic insight for post-vaccine myocarditis and pericarditis by deriving testable hypotheses backed by evidence derived from systems biology analyses. 

## 2. Materials and Methods

### 2.1. Dataset

Raw data files were downloaded in comma-separated value (CSV) files from the CDC website [13]. CDC WONDER online search tool was used to mine the VAERS [14]. We downloaded the 2021 VAERS public data set, which contained vaccine side reaction reports since 1990, and consisted of three separate data files: (1) VAERS data, (2) VAERS symptoms, and (3) VAERS vaccine. 

### 2.2. Databases

#### 2.2.1. VAERS

VAERS [14] was established in 1990, as a national early warning system to detect possible safety problems in U.S. licensed vaccines. It serves as is a post-marketing safety surveillance program, collecting information about adverse events (possible side effects) that occur after the administration of U.S. licensed vaccines. VAERS is co-managed by the U.S. Centers for Disease Control and Prevention (CDC) and the U.S. Food and Drug Administration (FDA). The VAERS data are updated weekly, and includes reports from 1990 onwards. 

#### 2.2.2. MetaCore^TM^

MetaCore^TM^ version 20.3 build 70,200 [15] from Clarivate Analytics is a database of manually composed ontologies mapped to canonical pathways and networks. We used this database for purposes of enrichment analysis in pathway maps. Pathway maps in MetaCore^TM^ are defined as subsets of functionally connected genes to describe a specific cellular process in a specific cellular context. Herein, the enrichment analysis is performed by examining the intersection between a gene list of myocarditis biomarkers extracted from MetaCore^TM^, and the prebuilt pathway maps in MetaCore^TM^ using the hypergeometric mean, which takes into account the number of objects in your dataset, the number of objects in the intersecting map and the number of objects in the entire database. This assessment returns a p-value that tells us the likelihood that the intersection between the gene signature and a particular map is obtained purely by chance. We set the p-value threshold at 0.05; rejecting all hypotheses/pathway maps that have enrichment p-values higher than 0.05.

### 2.3. Integrative Informatics Workflow

**Figure 1:**
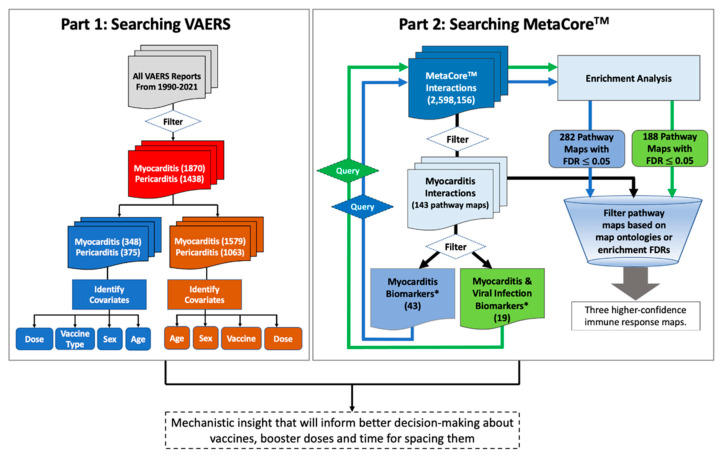
Workflow for mining post-vaccine adverse events (part 1) and understanding the underlying biology (part 2). Part 1 is a VAERS module to mine post-vaccine adverse events. The module starts by mining the VAERS database for post-vaccine adverse events then filtering by myocarditis and pericarditis adverse events, vaccine type, sex and age of the vaccinated individuals. Part 2 is a MetaCore^TM^ module to mine biological pathways associated with myocarditis and viral infection processes. This module starts by filtering Myocarditis pathway maps in MetaCore^TM^ followed by extracting all myocarditis biomarkers in addition to viral infection biomarkers and then using these biomarkers to conduct enrichment analysis for pathway maps in MetaCore^TM^. The identified pathway maps from the enrichment analysis plus pathway maps linked to myocarditis via any individual biomarkers (i.e., initial 143 pathway maps), were filtered based on FDR values and map ontologies to accept common pathway maps that have the smallest FDR values. *Biomarkers according to MetaCore^TM^ database.

We developed an informatics workflow (Figure 1), based on the methods developed by Hajjo et al. [16,17,18,19], to interrogate the network pharmacology of myocarditis/pericarditis to derive a testable mechanistic hypothesis that could explain post-vaccine cardiac adverse events. First, we started by searching VAERS using the following VAERS indexing terms for events: “myocarditis” and “pericarditis”. All reports were grouped according to age, sex, vaccine type, vaccine dose and event category. Cases missing patient age, sex, or type of vaccine were kept in the raw data files and labeled as “unknown”. All raw data files are deposited at the dataset repository. The identified sex, age groups and vaccine types and doses were used as input search terms for our informatics approach to derive a testable hypothesis regarding post-vaccine myocarditis. 

## 3. Results

Our search in VAERS databases indicated that myocarditis and pericarditis adverse events were not unique to COVID-19 vaccines; they were also reported after receiving smallpox, anthrax, typhoid, hepatitis B, influenza, and other vaccines to a smaller extent. However, myocarditis and pericarditis events were more prevalent after receiving mRNA COVID-19 vaccines than any other vaccine. This could be partly due to potentially smaller population sizes receiving the identified non-COVID-19 vaccines, or it may be due to other reasons. To understand this better, we applied a two-step informatics workflow (Figure 1) which helped in answering important questions regarding the most affected age groups, the most affected sex, the frequency of these adverse events after each vaccine dose, and biological mechanisms leading to myocarditis and pericarditis.

### 3.1. Post-COVID-19 Vaccine Myocarditis and Pericarditis Adverse Events

#### 3.1.1. Age Differences

**Figure 2:**
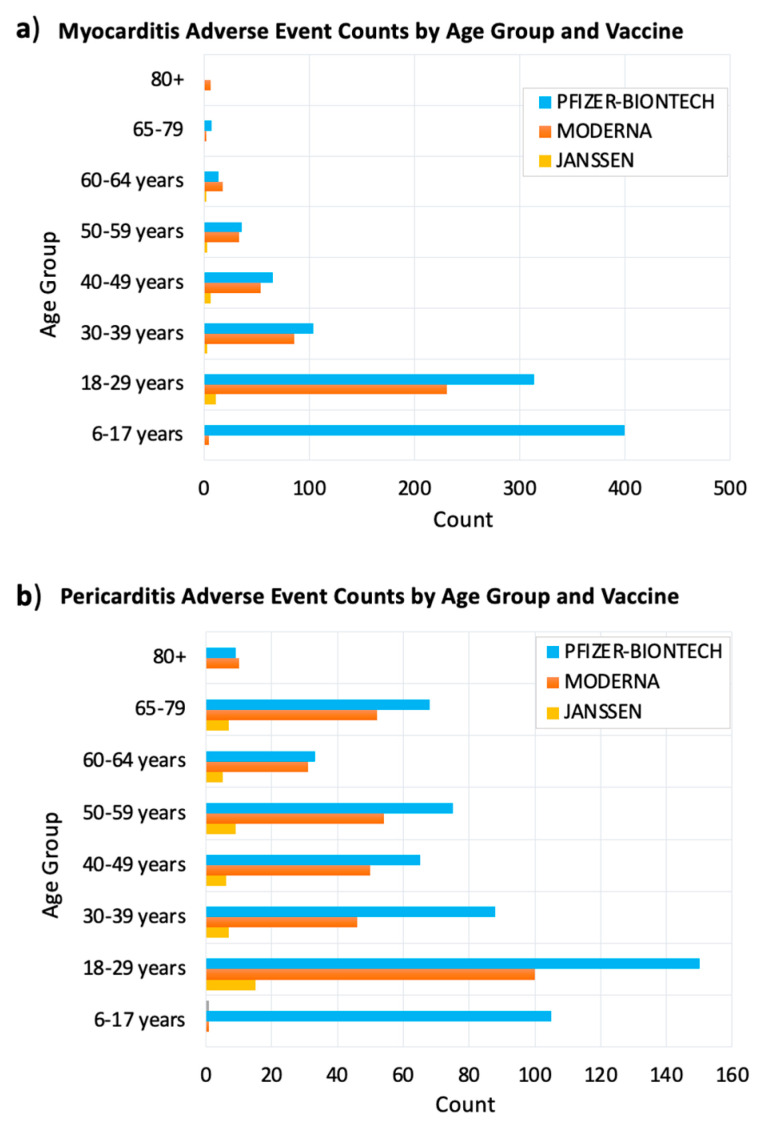
Reported post–COVID–19 vaccine myocarditis (**a**) and pericarditis (**b**) adverse events by age group and vaccine type. The x–axis shows the number of myocarditis side events after receiving COVID–19 vaccine. The y–axis shows the age groups of vaccine recipients. The bars were colored according to the vaccine received. VAERS reports were processed as of 2 September 2021.

We searched VAERS for myocarditis and pericarditis adverse events by COVID-19 vaccine and age group of the affected people. Our search resulted in 1579 myocarditis events and 1063 pericarditis events. The prevalence of myocarditis and pericarditis events in different age groups indicated that these two cardiac adverse events were predominant in young adults 18–29 years old. Myocarditis events were also prevalent in children 6–17 years old followed by adults 30–39 years old for myocarditis (Figure 2a). Pericarditis events were prevalent in adults 30–39 years old followed by children 6–17 years old (Figure 2b).

#### 3.1.2. Sex Differences

**Figure 3:**
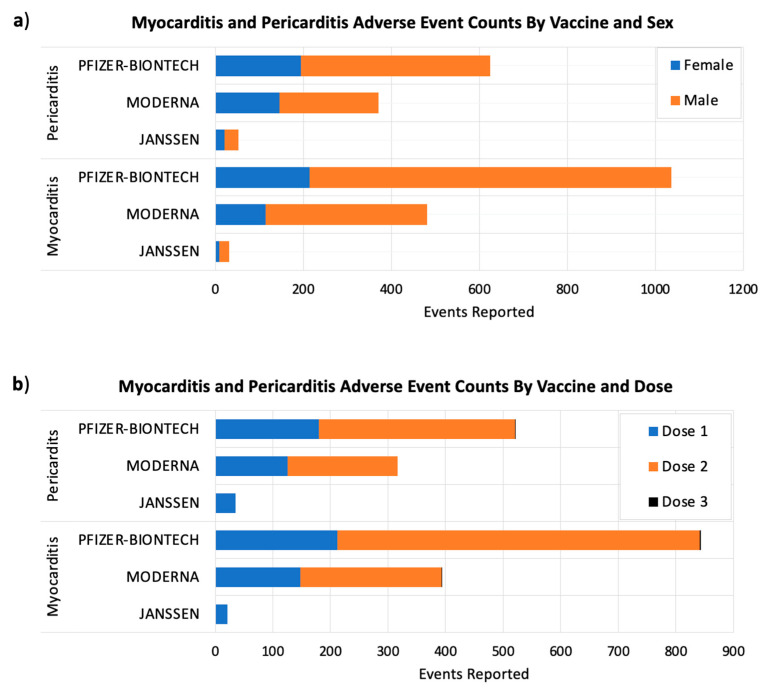
The number post-COVID-19 myocarditis and pericarditis adverse events by sex (**a**) and the administered vaccine dose (**b**). The x-axes show the adverse event counts for each COVID-19 vaccine shown on the y-axes. Considering VAERS reports processed as of 2 September 2021.

Our results showed large sex differences in the frequency of myocarditis and pericarditis adverse events occurring after receiving COVID-19 vaccines. The percentages of myocarditis and pericarditis adverse events in male subjects were 77% and 65% subsequently, in comparison to 22% myocarditis and 34% pericarditis adverse events in females (Figure 3a).

#### 3.1.3. Vaccine Dose Differences

Post-vaccine adverse events generally happen within six weeks of receiving a vaccine dose, and they could be more intense after booster doses, according to the U.S. Centers for Disease Control and Prevention (CDC) [20]. For this reason, the FDA requires each of the authorized COVID-19 vaccines to be studied for at least eight weeks after the final vaccine dose [20]. Currently, the majority of people around the world have taken either one or two vaccine doses. Our results showed that myocarditis and pericarditis events were more common after the second doses of Moderna and Pfizer/BIONTECH COVID-19 vaccines (Figure 3b). 

#### 3.1.4. Outcome Differences

To assess the severity of post-COVID-19 vaccine myocarditis and pericarditis events, we analyzed these adverse events by adverse event category. The VAERS database divided adverse events into multiple categories that range in severity from office visit to death (Supplementary Figure S1). These categories in order of increasing severity are office visit, emergency room, existing hospitalization prolonged, hospitalized, congenital anomaly/birth defect, permeant disability, life threatening, and death. Our results indicate that deaths remained fairly rare in comparison with hospitalizations and emergency room admissions. This confirms that these conditions are curable with proper treatment, and when they are diagnosed correctly.

### 3.2. Comparing Myocarditis and Pericarditis Events Occurring after Receiving COVID-19 versus Non-COVID-19 Vaccines

**Figure 4:**
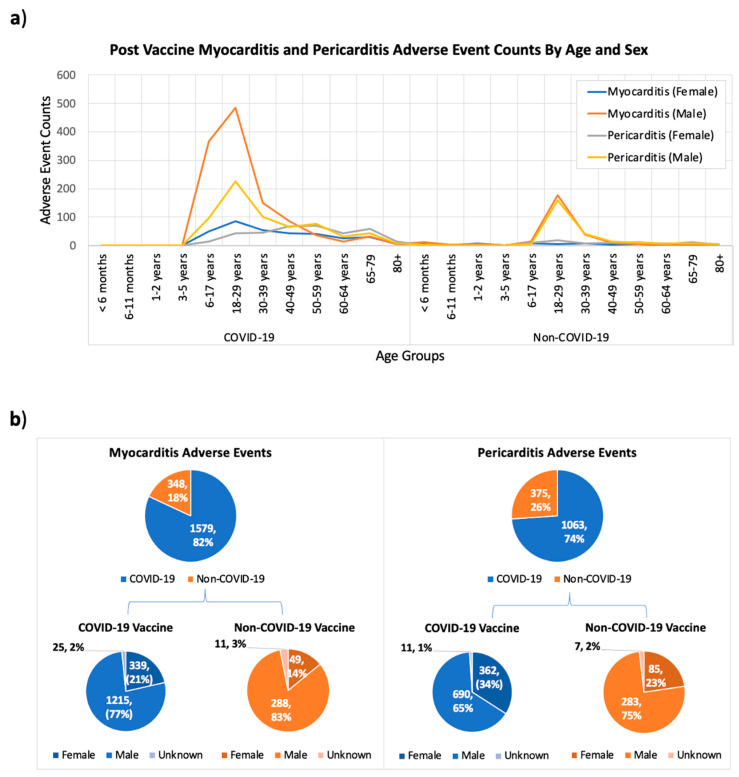
A comparison for post-vaccine myocarditis and pericarditis side effects between COVID-19 and Non-COVID-19 vaccines based on the age (**a**) and sex (**b**) of affected people. The x-axis in (**a**) shows the different age groups of vaccine recipients reporting adverse events for both COVID-19 and non-COVID-19 vaccines, and the y-axis shows the adverse even counts. Reports with unknown age records are not shown in (**a**). Considering VAERS reports processed as of 2 September 2021.

To check whether myocarditis and pericarditis events were unique to COVID-19 vaccines, or whether they were more serious after receiving a COVID-19 vaccine, we searched the VAERS database for all post-vaccine myocarditis and pericarditis events. Our results identified 1927 reported post-vaccine myocarditis events (348 events post non-COVID-19 and 1579 events post-COVID-19 vaccines), and 1438 pericarditis adverse events (375 events post non-COVID-19 and 1063 events post-COVID-19 vaccines) that were more common in young male adults (Figure 4a,b) indicating that myocarditis and pericarditis adverse events are not unique to COVID-19 vaccines, and can occur after receiving non-COVID-19 vaccines. However, the incidences of myocarditis and pericarditis were much higher after COVID-19 vaccines, and this could be partly due to the relatively larger number of people who received the COVID-19 vaccines.

Analyzing the distribution of myocarditis and pericarditis adverse events among different age groups indicated that these events were more predominant in young adults 18–29 years old, followed by adults 30–39 and children 7–16 years old (Figure 4a). We also found that these adverse events were more prevalent in males (%) than females (%) (Figure 4b). 

The top 10 vaccines associated with myocarditis and pericarditis are listed in Table 1. Myocarditis and pericarditis were more frequently reported after receiving the COVID-19 followed by smallpox and anthrax vaccines. It should be noted that smallpox, anthrax and typhoid vaccines are all live vaccines, and are only recommended in the United States to small vulnerable groups of the population in certain research jobs and travel situations according to CDC’s guidelines [21].

### 3.3. Identifying Causal Gene Associations with Myocarditis and Pericarditis

To understand the underlying biology of post-vaccine myocarditis and pericarditis, we applied a systems biology approach which starts by searching MetaCore^TM^ for myocarditis and pericarditis pathway maps to extract plausible high confidence mechanistic insight. In these searches we mined MetaCore^TM^ for genes and gene products on the DNA, RNA, and/or protein levels. We identified 41 biomarker genes for myocarditis which included the following higher confidence myocarditis biomarkers: (1) TNC (tenascin C) [22], (2) RNASE3 (ribonuclease A family member 3) [13] and (3) CD44 (Indian blood group antigen) [23]. Lab experiments found elevated protein levels (i.e., gene products) of these three genes in myocarditis [7,22]. We could not identify any causal gene associations with pericarditis, and there were not any MetaCore^TM^ pathway maps linked to pericarditis. Hence, this analysis will focus primarily on understanding myocarditis adverse events. 

We used two methods to link these 41 myocarditis biomarkers to pathway maps that can provide better mechanistic insight about the underlying biology. First, we mined 143 MetaCore^TM^ pathway maps that were linked to myocarditis biomarkers through at least one biomarker. Then we filtered these 143 maps based on map ontologies that had the following terms: “Virus”, “Viral”, “Antiviral”, and “Vaccine”. As a result, we identified two pathway maps that were related to antiviral actions: (1) Immune response map for the antiviral actions of interferons, and (2) immune response map for the role of PKR in stress-induced antiviral cell response. Filtering genes on each map based on established links to myocarditis highlighted the role of IFN-gamma as an important signaling molecule that turns on cardiac inflammation pathways. Example maps are shown in Supplementary Figures S2 and S3 (Supporting Information).

We also filtered the initial list of 41 myocarditis biomarkers, shown in Supplementary Table S1 (Supporting Information), by keeping overlapping biomarkers for myocarditis and antiviral action based on MetaCore^TM^ biomarker data; this filtering resulted in 19 biomarkers shown in Supplementary Table S2 (Supporting Information). Next, we performed enrichment analysis in MetaCore^TM^ to identify overrepresented pathways with FDR ≤ 0.05. We identified 282 pathways maps overrepresented for the 41-biomarker list, and 188 pathway maps for the 19-biomarker list. We identified three immune response pathway maps among top 10 most enriched pathways by the 19-biomarker list. All three maps were related to interferon-gamma signaling: (1) Immune response map for IFN-gamma signaling via MAPK (FDR = 3.661 × 10^−4^), (2) Immune response map for the induction of the antigen presentation machinery by IFN-gamma (FDR = 3.800 × 10^−4^), and (3) Immune response map for IFN-gamma signaling via PI3K and NF-kB (FDR = 4.269 × 10^−4^).

**Figure 5:**
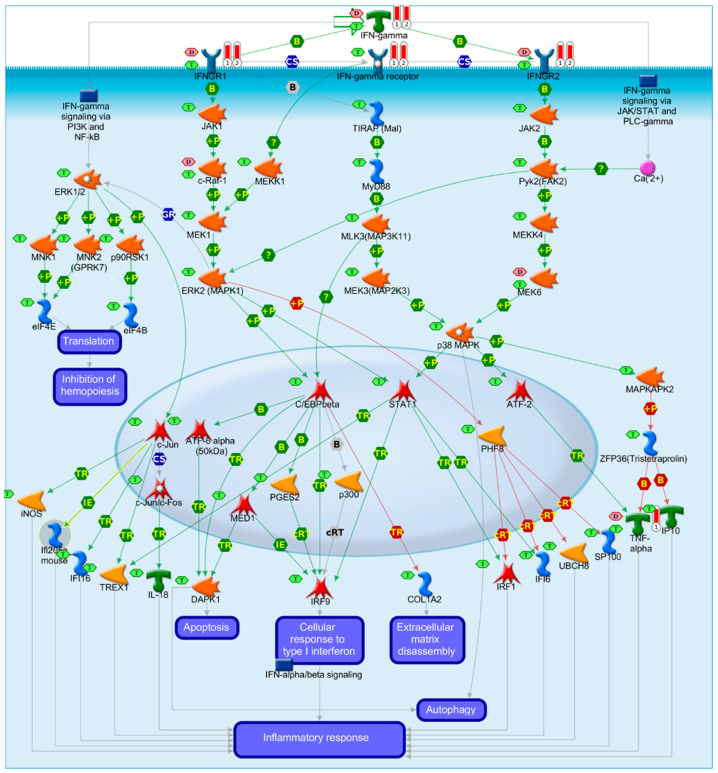
Immune response IFN-gamma signaling via MAPK and/or JAK/Stat pathways. B: binding; IE: influence on expression; TR: transcription regulation; red arrows for inhibition, green arrows for activation, gray arrows for unspecified action, violet text boxes for normal process, pink text boxes for pathological processes, blue text boxes for notes, starred network objects refer to groups or complex processes, pink hexagons indicate whether a network object is a biomarker for cardiomyopathies, and green hexagons indicate whether the network object is expressed in cardiovascular tissues, red thermometers indicate that the network object is a biomarker for myocarditis.

We also mined all pathway maps that had in their description the term “Myocarditis” and identified three pathway maps: (1) Immune response IFN-gamma signaling via MAPK, (2) Immune response IL-23 signaling pathway, and (3) A COVID-19 map for SARS-CoV-2 effects on infected tissues (Figure 5). Focusing on Immune response IFN-gamma signaling via MAPK that has been predicted by two different approaches that increased the confidence in this pathway map, highlighted two key biomarkers for myocarditis and antiviral immune processes: IFN-gamma and TNF-alpha. 

This result signifies the role of IFN-gamma signaling in post-vaccine inflammatory adverse events that may affect many organs including the heart. IFN-gamma signaling is an immune effector for various vaccines, and it is not unique for COVID-19 vaccines [24,25,26]. The type of the vaccine exerts a direct influence on the type of immune effectors and magnitude of mounted immune response that mediate protective efficacy of vaccines [27]. In fact, inflammatory cytokines have been proposed recently to mediate post-vaccine allergic reactions [28].

IFN-gamma has been generally associated with the promotion of T_H_1 immune response [29,30] which is strongly triggered in response to live vaccines versus ‘non-live’ vaccines [31]. In this regard, mRNA vaccines seem to emulate the effects of live vaccines which makes sense because viral mRNA triggers the activation of the innate immune system through multiple pathogen-associated signals. In fact, stronger T_H_1 responses imply higher intensity of innate responses and more prolonged antigen persistence generally which results in higher antibody (Ab) responses to live versus ‘non-live’ vaccines [27]. 

This situation is very similar to that occurring after a natural infection where dendritic cells (DCs) are activated at multiple sites, migrate toward the corresponding draining lymph nodes, and launch multiple foci of T- and B-cell activation. This sequence provides a second explanation of the generally higher immunogenicity of live versus “non-live” vaccines.

For pericarditis, we identified PRG4 gene (proteoglycan 4) as the only gene associated with pericarditis according to MetaCore methods and data in the biomedical literature. This association between PRG4 and pericarditis is based on a mutational study indicating that mutations in PRG4 gene on chromosome 1 result in camptodactyly arthropathy-coxa vara-pericarditis syndrome [32]. To check if there are any direct links between PRG4 and IFN-gamma, we attempted to build a “direct interactions” network but no links were identified. Therefore, the “auto expand” algorithm was used to derive sub-networks around PGR4 and IFN-gamma. Then, the derived sub-networks keep expanding until they intersect. The resulting larger network (Supplementary Figure S4) highlighted a link between PGR4 and IFN-gamma via TCF/LEF protein group including LEF1 (lymphoid enhancer binding factor 1), TCF7 (Transcription factor 7) also known as TCF1 (T-cell-specific transcription factor 1) and TCF7L2 (transcription factor 7 like 2) also known as TCF4 (T-cell-specific transcription factor 4).

## 4. Discussion

In an effort to understand post-vaccine myocarditis and pericarditis side effects that have been linked to mRNA vaccines lately, according to a statement from the CDC [13], we applied an informatics workflow to mine the VAERS database for post-vaccine myocarditis, followed by a systems biology analysis that relied on mining specialized chemogenomics databases for genes, proteins and chemicals that are linked to myocarditis. There were three COVID-19 vaccines included in VAERS database (i.e., vaccines approved by the U.S. FDA) including Pfizer/BioNTech, Moderna and Janssen. These vaccines were prepared by two technologies: (1) mRNA encoding the SAR-CoV-2 spike (S) protein encapsulated in lipid nanoparticle such as the two vaccines by Pfizer/BioNTech and Moderna, and (2) adenovirus (AdV) vectors encoding the S protein such as Janssen’s vaccine. The different vaccine preparations enter dendritic cells (DCs) at site of injection or within lymph nodes, resulting in production of S protein [33].

Our results indicated that post-COVID-19 vaccine myocarditis and pericarditis adverse events were more prevalent in young males 18-29 years old. Actually, a recent opinion [34] in the journal Pediatric Reports raised concerns about mRNA vaccines for adolescents, and questioned why cardiac adverse events have higher incidence in male adolescents after receiving the second dose of the vaccine and what mechanisms are contributing to these adverse events. Our data also showed that the number of myocarditis and pericarditis adverse events was higher for Pfizer/BioNTech vaccine followed by Moderna’s and then Janssen’s (based on the available data described herein). However, it should be noted that the Pfizer/BioNTech and Moderna vaccines were more widely administered in the United States than Janssen’s. Therefore, we cannot conclude based on the data described here in that myocarditis was more frequent after receiving mRNA vaccines. 

We also found evidence in the biomedical literature documenting cardiac side effects after receiving smallpox and anthrax vaccines [4,35,36,37,38,39,40,41,42]. There is also clinical evidence for developing myocarditis in 59% smallpox and 23% anthrax vaccinated individuals [4]. In fact, vaccinated individuals from Europe and Australia reported more post smallpox vaccine cardiac complications suggesting that the vaccine strains or preparations used there might been more immunologically reactive in comparison to the vaccine strains used in the United States which is knowns as the New York City Board of Health (NYCBOH) strain [43]. Other clinical studies found evidence for post-vaccine myocarditis after receiving typhoid [44] and hepatitis B vaccines [45]. 

Our systems biology results highlighted central signaling roles for IFN-gamma and TNF-alpha in both myocarditis and viral disease maps. Finally, combing the knowledge from searching both VAERS and MetaCoreTM enabled the exploration of age and sex differences of post-vaccine myocarditis. We found evidence in the biomedical literature indicating gender and age differences in many immunological factors. The sex differences in the levels of proinflammatory cytokines, including TNF-alpha and IFN-gamma, increase at puberty and then wane later in life suggesting hormonal effects. This goes in concert with our findings about the prevalence of post-vaccine myocarditis in the age group in adolescents and young adults (Figure 2, Figure 3a and Figure 4). Furthermore, Aomatsu et al. reported on the gender differences in in TNF-alpha production in human neutrophils stimulated by lipopolysaccharide (LPS) or IFN-gamma plus LPS [46]. It was suggested that the lower sensitivity of female neutrophils to LPS and IFN-gamma, was due to the anti-inflammatory effects of estradiol on vascular endothelial cells and monocytes/macrophages [46]. However, for immune response factors the sex difference remains constant from birth to old age (for example higher numbers of CD4+ T cells and higher rations of CD4/CD8 T have been reported in females at all age groups) [46]. 

To fathom post-vaccine myocarditis, it is important to understand that post-vaccine side effects (e.g., injection site pain, fever, chills, swelling fatigue, headache, muscle pain, joints pain, and others) [14,47] are caused by the secondary enhancement of the inflammatory response which often results from short-term changes to innate cells such as macrophages through ‘trained immunity’ [48], and/or from the activation of memory T cells and B cells generated from the initial injection [33]. There is also evidence that systemic vaccine side effects can be augmented with the second vaccine doses [49] due to an amplified release of type 1 interferon which plays a central role in amplifying T-cell memory and B-cell differentiation and survival. This implies that post-vaccine inflammation after receiving booster doses, can further promote the generation and perpetuation of long-term immunological memory [33,50,51].

We found evidence that IFN-gamma is also key component of the host defense to viral infection [52,53,54,55] that is also elicited in response to viral vaccines [56]. MRNA and viral vector vaccines trigger spike protein synthesis inside human cells which can then be presented on cell surfaces to cytotoxic T cells by the major histocompatibility complex (MHC) MHC class I and MHC class II molecules, leading to virus-specific recognition and killing of infected or vaccinated cells. Interferons alpha, beta and gamma are required to induce MHC class I expression, while MHC class II transactivator factor (CIITA) is the master regulator of MHC class II expression and it is efficiently induced by IFN-gamma. However, most cell types do not express basal CIITA, and its expression is induced by IFN-gamma [57,58]. In myocarditis, T lymphocytes (T cells), have been reported to home the myocardium and to acutely reduce left ventricular (LV) function and this has been associated with increased levels of circulating cytokines, such as TNF-α which led to the prioritization of proinflammatory cytokines as markers of myocarditis and heart failure. It is also known that antigen presentation by endothelial cells may initiate rapid and localized ‘memory’ immune responses in peripheral tissues. Antigens displayed on major histocompatibility complex (MHC) molecules on the surface of endothelial cells can then be recognized by T-cell receptors on circulating effector memory T cells triggering both trans-endothelial migration and activation [59]. 

Type I interferons are key drivers for the antiviral state in non-immune cells and they orchestrate antiviral immune responses through: (i) the inhibition of viral replication in infected cells during the innate stage of the immune response; (ii) the activation and enhancement of antigen presentation in the “early induced” immune response, and (iii) the generation of the adaptive immune response through direct and indirect actions on T and B cells that constitute the memory response (reviewed in References [50,60]). TH1 response, which triggers IFN-gamma expression that in turn activates CTLs and NK cells. From a homeostatic point of view, prolonged effector function of these cells may lead to excessive cytotoxicity and other detrimental effects resulting in host tissue damage [50,61]. Additional support for the identified role of IFN-gamma in myocarditis came from a recent Nature publication by Arunachalam et al. [62] confirming that Pfizer/BioNTech mRNA vaccine induced a heightened innate immune response after secondary immunization relative to primary immunization implicating the overexpression of IFN-gamma in driving innate and antiviral responses after the booster.

It should be noted that this study has a few limitations. First, while VAERS is a valuable source for monitoring vaccine adverse effects, reports from VAERS alone may not be conclusive to establish a causal relationship between an adverse event and a vaccine use. Due to the voluntary nature of VAERS reports, the information provided about an adverse effect can be imperfect, imprecise, coincidental, or unconfirmed, limiting the scientific use of such reports [14]. Though the CDC and FDA usually follow up on all serious adverse event reports to obtain additional medical, laboratory, death certificates, and/or autopsy records, nonetheless changes in information based on further investigations of serious adverse events may not be reflected on VAERS public data. Secondly, bioinformatics techniques relying on gene expression, pathway over-representation and network biology have the following limitations and biases that we reviewed by Hajjo and Tropsha elsewhere [17]. While we were finalizing this manuscript, we learned that the CDC is planning an ‘emergency meeting’ on rare post-COVID-19 vaccine heart inflammation [63].

Based on research findings presented here, we suggest that increasing the time interval between the first and second vaccines doses could potentially reduce or eliminate myocarditis adverse events by reducing the chances of unfavorable synergism on interferon-gamma secretion resulting from the successive waves of primary responses. We also prioritize the use of glucocorticoids (e.g., dexamethasone) for post-COVID-19 vaccine myocarditis for the following reasons: (1) glucocorticoids has been used successfully for the treatment of acute and chronic myocarditis, including viral myocarditis [27,63,64,65,66,67,68,69], (2) glucocorticoids lead to the inhibition of IFN-gamma by regulating STAT1 expression [19], (3) dexamethasone is one of the drugs that reduces COVID-19 mortality. 

## 5. Conclusions

Our study suggests that myocarditis adverse events are linked to vaccine type (i.e., COVID or non-COVID), technology (e.g., mRNA or inactivated), age and sex of the vaccinated individuals. Based on VAERS data, post-vaccine myocarditis was most frequently reported for live vaccines (e.g., smallpox, anthrax and some typhoid vaccines), or vaccines that behave like live vaccines such as mRNA and possibly viral vector vaccines (e.g., COVID-19 vaccines) versus inactivated vaccines. The suggested mechanism of post-vaccine-myocarditis involves IFN-gamma signaling and T_H_1 immune responses following vaccination, which can be modulated by increasing the time interval between first vaccine doses and following boaster doses, or by the use of glucorticoids. Herein we applied a systems biology informatics approach for studying post-vaccine myocarditis adverse events which resulted in testable hypotheses explaining the biological mechanisms leading to these events and led the prioritization of pharmaceutical and non-pharmaceutical mitigation strategies. This approach can be applied for conducting similar analyses involving other side effects or other vaccines, hence, our workflow stands ready for following up on any alarming report from the CDC. 

## Figures and Tables

**Table 1:** The counts and percentages of reported myocarditis and pericarditis adverse events associated with top 10 vaccine types ranked based on the number of events reported as of 2 September 2021.

Myocarditis Adverse Events				
Rank	Vaccine Type	Vaccine Type Code *	Events Reported	Percent
1	COVID-19 VACCINE	COVID19	1579	87.19%
2	SMALLPOX VACCINE	SMALL	223	12.31%
3	ANTHRAX VACCINE	ANTH	63	3.48%
4	TYPHOID VACCINE	TYP	29	1.60%
5	HEPATITIS B VACCINE	HEP	26	1.44%
6	INFLUENZA VIRUS VACCINE, TRIVALENT (INJECTED)	FLU3(SEASONAL)	20	1.10%
7	HAEMOPHILUS B CONJUGATE VACCINE	HIBV	18	0.99%
8	INFLUENZA VIRUS VACCINE, NO BRAND NAME	FLUX(SEASONAL)	18	0.99%
9	HEPATITIS A	HEPA	16	0.88%
10	VARIVAX-VARICELLA VIRUS LIVE	VARCEL	16	0.88%
Pericarditis Adverse Events				
Rank	Vaccine Type	Vaccine Type Code	Events Reported	Percent
1	COVID-19 VACCINE	COVID19	1063	79.15%
2	SMALLPOX VACCINE	SMALL	200	14.89%
3	ANTHRAX VACCINE	ANTH	63	4.69%
4	INFLUENZA VIRUS VACCINE, TRIVALENT (INJECTED)	FLU3(SEASONAL)	35	2.61%
5	TYPHOID VACCINE	TYP	33	2.46%
6	ZOSTER VACCINE	VARZOS	29	2.16%
7	HEPATITIS B VACCINE	HEP	20	1.49%
8	INFLUENZA VIRUS VACCINE, NO BRAND NAME	FLUX(SEASONAL)	19	1.41%
9	INFLUENZA VIRUS VACCINE, TRIVALENT (INTRANASAL SPRAY)	FLUN3(SEASONAL)	12	0.89%
10	HUMAN PAPILLOMAVIRUS (TYPES 6, 11, 16, 18) RECOMBINANT VACCINE	HPV4	10	0.74%

* According to the VAERS databases.

## Data Availability

Biomarkers data used in this study are available at *Vaccines* online. All VAERS raw data files are available on GitHub (https://github.com/rhajjo/Myocarditis-Pericarditis).

## Supplementary Materials

The following are available online at https://www.mdpi.com/article/10.3390/vaccines9101186/s1, Table S1: List of myocarditis biomarkers, Table S2: The list of common biomarkers for myocarditis and virus infection, Figure S1: The distribution of post-COVID-19 vaccine myocarditis and pericarditis adverse events by category, Figure S2: Immune response map for the role of PKR in stress-induced antiviral cell response, Figure S3: Immune response map for the antiviral actions of Interferons, Figure S4: Network connecting IFN-gamma with PRG4.
