# Supplementary material for: Shedding the Light on Post-Vaccine Myocarditis and Pericarditis in COVID-19 and Non-COVID-19 Vaccine Recipients
Source: Vaccines (Basel). 2021 Oct 15;9(10):1186. doi: 10.3390/vaccines9101186 (PMC8541143; doi:10.3390/vaccines9101186)
Supplement: Supplementary file 1 [file vaccines-09-01186-s001.zip › vaccines-1411296-supplementary.pdf]

Shedding the Light on Post-vaccine Myocarditis and Pericarditis in COVID-19 and Non-COVID-19 Vaccine Recipients

Rima Hajjo[1,2,3*], Dima A. Sabbah[1], Sanaa K. Bardaweel[4], Alexander Tropsha[2]

[1]Department of Pharmacy, Faculty of Pharmacy, Al-Zaytoonah University of Jordan, P.O. Box 130 Amman 11733 Jordan.

[2]Laboratory for Molecular Modeling, Division of Chemical Biology and Medicinal Chemistry, Eshelman School of Pharmacy, The University of North Carlina at Chapel Hill.

[3]National Center for Epidemics and Communicable Disease Control, Amman, Jordan.

[4]Department of Pharmaceutical Sciences, School of Pharmacy, University of Jordan, Amman 11942, Jordan

***Correspondence**

Rima Hajjo. E-mail: rhajjo@gmail.com; r.hajjo@zuj.edu.jo

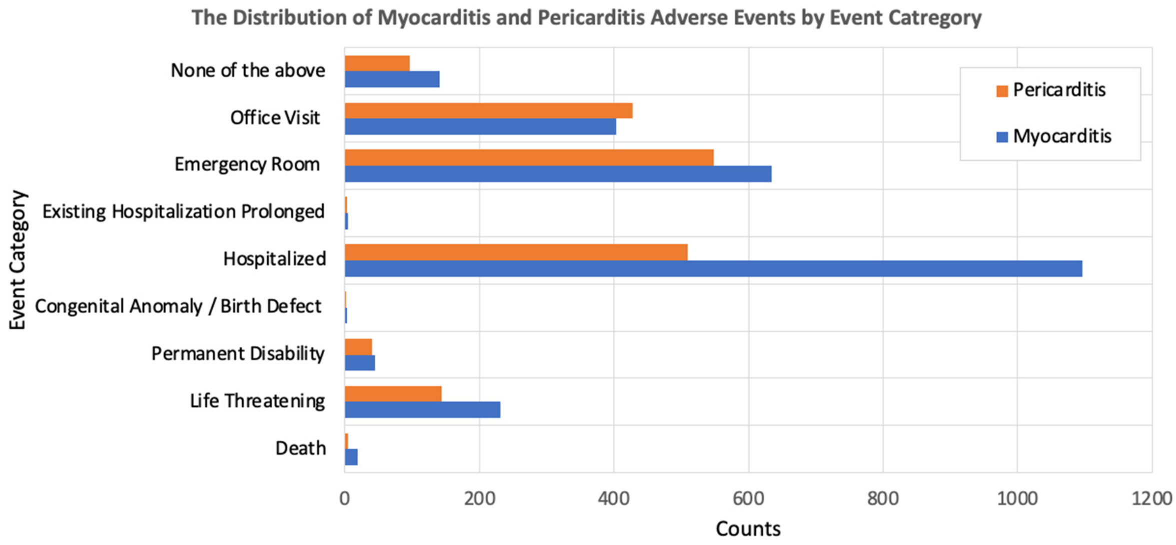

Figure S1. The distribution of post COVID-19 vaccine myocarditis and pericarditis adverse events by category.

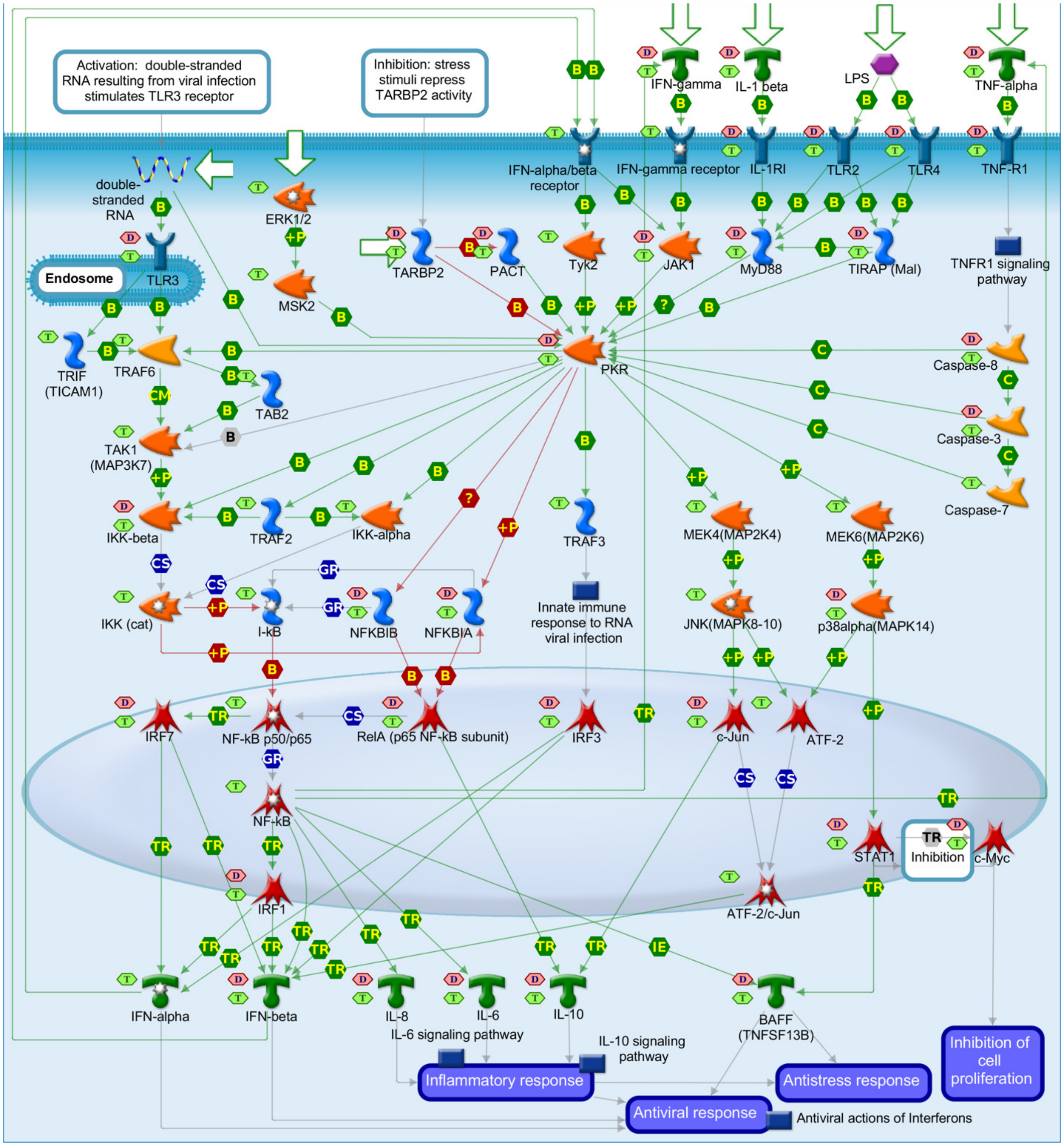

Figure S2. Immune response map for the role of PKR in stress-induced antiviral cell response. B: binding; IE: influence on expression; TR: transcription regulation; red arrows for inhibition, green arrows for activation, grey arrows for unspecified action, violet text boxes for normal process, pink text boxes for pathological processes, blue text boxes for

notes, starred network objects refer to groups or complex processes, pink hexagons indicate whether a network object is a biomarker for cardiomyopathies, and green hexagons indicate whether the network object is expressed in cardiovascular tissues, red thermometers indicate that the network object is a biomarker for myocarditis.

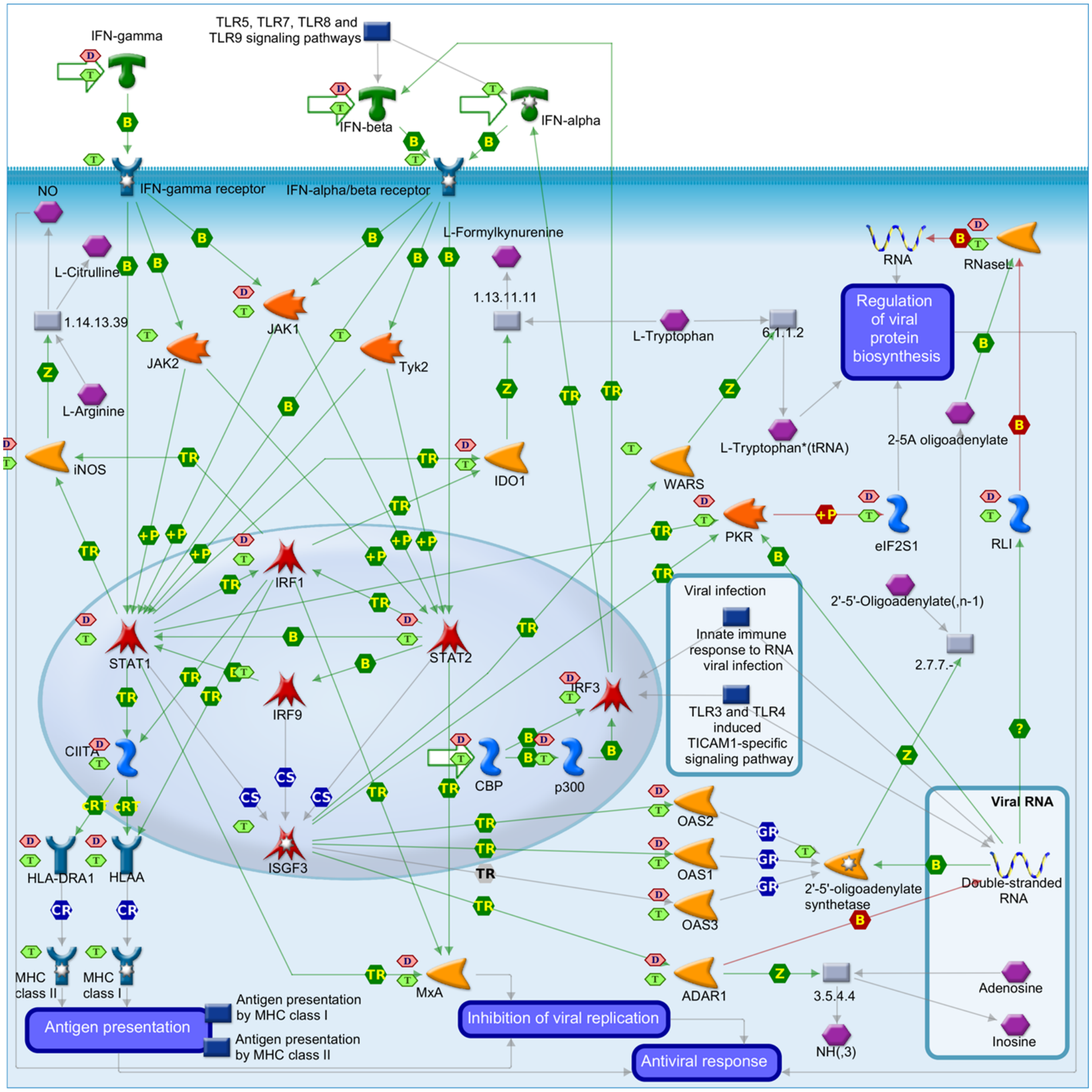

Figure S3. Immune response map for the antiviral actions of Interferons. B: binding; IE: influence on expression; TR: transcription regulation; red arrows for inhibition, green arrows for activation, grey arrows for unspecified action, violet text boxes for normal process, pink text boxes for pathological processes, blue text boxes for notes, starred network objects refer to groups or complex processes, pink hexagons indicate whether a

network object is a biomarker for cardiomyopathies, and green hexagons indicate whether the network object is expressed in cardiovascular tissues, red thermometers indicate that the network object is a biomarker for myocarditis.

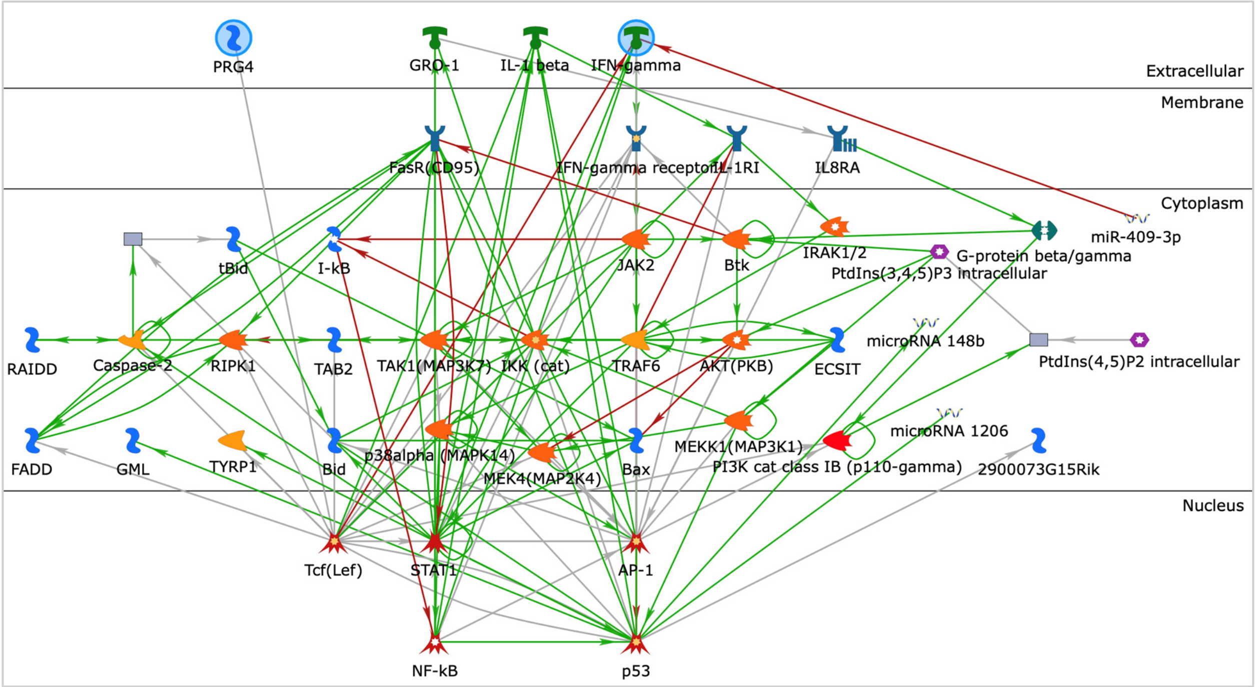

Figure S4. Network connecting IFN-gamma with PRG4. The network was generated using the auto-expand algorithm in MetaCoreTM and canonical pathways, with maximum number of nodes allowed = 50. Red arrows for inhibition, green arrows for activation, and grey arrows for unspecified action.
